# Metabolic network reconstruction of *Euglena gracilis*: Current state, challenges, and applications

**DOI:** 10.3389/fmicb.2023.1143770

**Published:** 2023-03-02

**Authors:** Sahutchai Inwongwan, Jeeraporn Pekkoh, Chayakorn Pumas, Pachara Sattayawat

**Affiliations:** ^1^Department of Biology, Faculty of Science, Chiang Mai University, Chiang Mai, Thailand; ^2^Research Center of Microbial Diversity and Sustainable Utilizations, Faculty of Science, Chiang Mai University, Chiang Mai, Thailand; ^3^Research Center in Bioresources for Agriculture, Industry and Medicine, Chiang Mai University, Chiang Mai, Thailand

**Keywords:** metabolic network reconstruction, genome-scale metabolic model, metabolic network modelling, metabolic flux analysis, *Euglena gracilis*

## Abstract

A metabolic model, representing all biochemical reactions in a cell, is a prerequisite for several approaches in systems biology used to explore the metabolic phenotype of an organism. Despite the use of *Euglena* in diverse industrial applications and as a biological model, there is limited understanding of its metabolic network capacity. The unavailability of the completed genome data and the highly complex evolution of *Euglena* are significant obstacles to the reconstruction and analysis of its genome-scale metabolic model. In this mini-review, we discuss the current state and challenges of metabolic network reconstruction in *Euglena gracilis*. We have collated and present the available relevant data for the metabolic network reconstruction of *E. gracilis*, which could be used to improve the quality of the metabolic model of *E. gracilis*. Furthermore, we deliver the potential applications of the model in metabolic engineering. Altogether, it is supposed that this mini-review would facilitate the investigation of metabolic networks in *Euglena* and further lay out a direction for model-assisted metabolic engineering.

## 1. Introduction

*Euglena gracilis* is a photosynthetic protist with a long history of being a model organism in biological studies. It can grow in autotrophic, heterotrophic or mixotrophic conditions (Takeyama et al., 1997), aerobically or anaerobically, and over a wide range of pH (Yamane et al., 2001). *E. gracilis* has been considered as a potential dietary supplement due to its capacity to produce various bioactive compounds and is a useful source of proteins, polyunsaturated fatty acids, vitamin A, vitamin C and vitamin E (Korn, 1964; Takeyama et al., 1997; Kusmic et al., 1998; Ogbonna et al., 1998; Barsanti et al., 2000; Gissibl et al., 2019). Moreover, *E. gracilis* accumulates storage carbohydrate in the form of β-1,3-glucan, which can make up to 85% of cell dry weight (Inui et al., 1982), called paramylon. Several medicinal properties of paramylon have been reported including antiviral and immunomodulatory effects (El Khoury et al., 2012; Murphy et al., 2020). Even though, this organism has long been a focus of research for its diverse industrial applications, it is confounding that the understanding of its metabolic capacity is still highly limited. Moreover, the analysis of the *Euglena* genome is still incomplete and restricted by its size and complexity (Ebenezer et al., 2019) arisen from multiple secondary endosymbiosis events during its evolution (Novák Vanclová et al., 2020).

**Figure 1:**
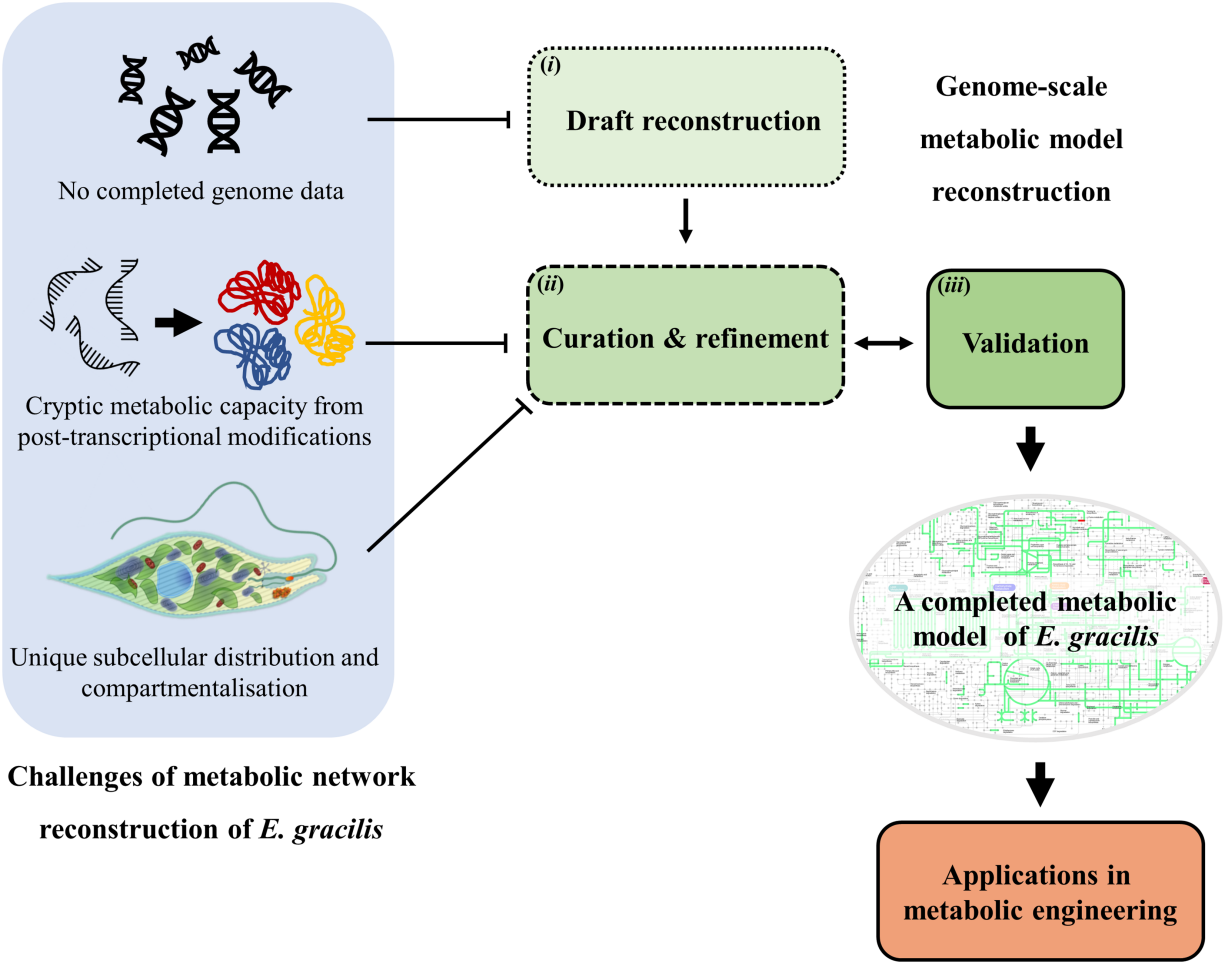
A schematic diagram representing the overview of this mini-review including steps of metabolic model reconstruction using genome data: (i) Draft reconstruction (ii) curation and refinement (iii) Validation of the model and challenges encountered when reconstructing the genome-scale model of *Euglena gracilis*. Potential applications of the network model are also discussed.

*E. gracilis* has received significant attention as a potential cell factory due to their ability to produce a diverse array of valuable chemicals. Efforts are currently focused on improving their ability to produce these chemicals in a cost-effective manner. Metabolic network reconstruction is an approach used to identify and characterise metabolic pathways present inside of an organism, which allow understandings of the metabolism inside of the cell. These reconstructed metabolic networks can then be used in several aspects. For example, from a metabolic engineering perspective, the comprehensive understanding of the metabolic pathways within a cell enables the rational selection of engineering targets. Interestingly, this approach has been well-explored in other model organisms, yet the development has been slow in the case of *E. gracilis*. Therefore, in this mini-review, the current state of the reconstruction of the metabolic network of *E. gracilis* is established, providing an overview of its metabolic network and highlighting the unique features of the network. In addition, we emphasise the challenges for reconstructing the network model of *E. gracilis* and deliver the available data that could be exploited to improve the completion of the metabolic model. Furthermore, possible applications of the model in metabolic engineering for the production of valuable products are also discussed. An overview of the content is presented in Figure 1.

## 2. Current state of the reconstruction of the metabolic network of *Euglena gracilis*

The exceptionally versatile metabolic capacity of *E. gracilis* is reflected in its broad range of growth conditions and substrate utilisation. The central pathways have been characterised, including glycolysis, gluconeogenesis, the tricarboxylic acid cycle (TCA), the pentose phosphate pathway (PPP), and the metabolism of lipids and amino acids (Inwongwan et al., 2019). The genome of *Euglena* was estimated to be approximately 500 Mb in size (Ebenezer et al., 2019) and has not been completely analysed. A complete sequence of *E. gracilis* chloroplast genome was published in 1993 (Hallick et al., 1993). The transcriptomic analysis indicates the presence of the biosynthesis pathways of carotenoids, thylakoid glycolipids, fatty acids, and isoprenoids. It also demonstrates the capacity to utilise the pathways for vitamin C, vitamin E, and glutathione metabolism to respond to stresses and to produce multifunctional polydomain proteins related to fungi and bacteria (O’Neill et al., 2015b). A study of the regulatory system of wax-ester metabolism under anaerobic conditions of *E. gracilis* using the comparative transcriptomic approach (Yoshida et al., 2016) reported that the differentially expressed genes from aerobic and anaerobic conditions were not involved in wax-ester metabolism, indicating that the metabolic pathways involved in wax-ester biosynthesis were regulated at the post-transcriptional level. In addition, the following published transcriptome data of *E. gracilis* also suggested that gene regulation in euglenozoans is not primarily controlled at the transcriptional level (Cordoba et al., 2021). The study of the mitochondrial genome of *E. gracilis* revealed the flexible mitochondrial metabolisms (Dobáková et al., 2015; Ebenezer et al., 2019), the mitochondria can produce energy under either aerobic or anaerobic conditions, and efficiently utilise a diverse set of organic respiratory substrates facilitated by the unique subcellular localisation of the metabolic pathways in mitochondria, such as glyoxylate cycle and alcohol oxidisation (Inwongwan et al., 2019). Altogether, the transcriptomic analyses of *E. gracilis* emphasise its versatile metabolic capacity and the regulation at post-transcriptional level (Yoshida et al., 2016; O’Neill et al., 2015b). Thus, using only the transcriptomic approach might not be sufficient to understand how *E. gracilis* responds to various conditions. The *E. gracilis* plastid proteome indicates the function of photosynthesis and demonstrates the core plastid metabolic pathways (Ebenezer et al., 2019; Novák Vanclová et al., 2020); however, there is no evidence of the presence of oxidative pentose phosphate pathway in its secondary chloroplast. Moreover, metabolomic analysis of *E. gracilis* reported changes in pathways used in response to environmental stresses (He et al., 2021). Nevertheless, the metabolome is not able to elucidate the systemic metabolic operation of the whole metabolic network.

Transcript sequences and topology gap filling were used to attempt to reconstruct the metabolic network of *E. mutabilis* (Halter et al., 2015; Prigent et al., 2017). This network model was reported to be incomplete as it could not simulate the growth of *E. mutabilis* in the dark (Prigent et al., 2017), despite the capacity of this species to do so. A draft of metabolic network model of *E. gracilis* was constructed specifically to study the heterotrophic metabolism of various kinds of carbon substrates which mainly includes the operation of the central metabolic pathways (Inwongwan, 2021), most of the peripheral pathways and reactions of photosynthesis were not extensively curated or tested. There have not been any other reports of a completed reconstructed metabolic network of *E. gracilis*.

## 3. Challenges of metabolic network reconstruction of *Euglena gracilis*

Genome sequence is generally a prerequisite for reconstructing the metabolic network of an organism (Thiele and Palsson, 2010). Genome-scale metabolic model (GEM) is a mathematical model consists of all metabolic reactions in a cell and their stoichiometries generally based on genome data, which is able to quantify the genotype–phenotype relationships (Fang et al., 2020). It has become a powerful tool in systems biology to study responsive metabolic phenotypes and optimise the production of targeted metabolites in metabolic engineering. Steps of reconstructing GEM start with drafting a reconstruction from the annotated genome data, and then, the draft model is manually curated and refined based on the physiological and/or biochemical evidences to increase the precision and accuracy. Subsequently, the experimental data including biomass composition, media composition and consumption rate, growth characteristics and other environmental factors in the mathematical model format are integrated into the model. After completing the draft reconstruction, network verification, debugging and gap filling are performed. The last step is evaluation and validation of the model depending on the objective of the reconstruction (Thiele and Palsson, 2010). This bottom-up reconstruction procedure applies well with the model organisms due to the accessible organism-specific genome and biochemical data. Several automated tools for generating GEM were developed based on the available databases of the model species, and GEM modelling are particularly well-developed in prokaryotes. However, the metabolic network reconstruction processes are not as straightforward for eukaryotic and non-model species (Yan and Fong, 2017; Hanna et al., 2020), especially for ones with complex evolution causing the diverse and cryptic metabolisms, unique subcellular localisation of pathways and organelles, and without an available completed set of genome data, like *Euglena*. Allegedly, reconstructing the GEM of *E. gracilis* comes with several challenges that need to be overcome to enable the generation of a descriptive GEM. Here, we have listed the main challenges encountered over the years.

### 3.1. No completed genome data available

Genomic analysis can lead to an investigation of the organism in numerous aspects (Griffiths et al., 2015). The conventional GEM reconstruction requires the completed analysed genome data as the initiating material for drafting the reconstruction as annotated genomes provides the absolute genetic and metabolic capacity of the network. Even though there are several computational automate tools to generate GEMs from genome data, the ability to produce the high-quality GEM and the application of these tools are usually restricted to the well-define organisms, partially due to a lack of complete annotated genome sequence and available related data (Passi et al., 2021). Despite the history of *E. gracilis* in biochemical and physiological research, the genome of *E. gracilis* has not yet to be fully analysed. Its complex evolution results in a massive genome size with a chimeric and convoluted structure, obstructing the assembly and analysis of the genome (Ebenezer et al., 2019; O’Neill et al., 2015a).

### 3.2. The cryptic metabolic capacity from post-transcriptional modifications

Several transcriptomic analyses underline that *E. gracilis* metabolic phenotypes can be significantly controlled by the post-transcription modification and regulation processes (O’Neill et al., 2015b; Yoshida et al., 2016; Ebenezer et al., 2019; Cordoba et al., 2021), demonstrating the cryptic but great metabolic capacity and complex cellular regulatory mechanisms. The high level of involvement of post-transcriptional modification creates a great challenge in curating and simulating the GEM for the specific conditions of interest. Regardless of the unavailability of the complete genome data, the transcriptomes would be insufficient to indicate the responsive metabolic mechanisms to the condition of interest or to depict the wholistic metabolic capacity of *E. gracilis*. Without this information, some significant metabolic processes in the network could be missed to identify.

### 3.3. Unique subcellular distribution and compartmentalisation of the metabolic pathways

Reconstruction of GEM of eukaryotes are challenging by the size of genomes and the multitude of cellular compartments (Thiele and Palsson, 2010). The compartmentation of metabolism in eukaryotes, especially ones with plastid (s), complicates the structure on the GEM due to the uncertainties of the distribution of the specific enzymes (Kruger and Ratcliffe, 2015). Locating subcellular locations of proteins and integrating them into the GEMs of eukaryotes have been one of the crucial steps to generate the accurate GEM. The subcellular localisation of central metabolic pathways of *E. gracilis* was previously reported, demonstrating the specific subcellular pathway distribution and the ambiguity for the function of its secondary chloroplast in heterotrophic metabolism. The study also emphasises the difficulty in predicting the subcellular location of *E. gracilis* enzymes from peptide sequences as the transportation into *E. gracilis* chloroplasts is not fully understood or well-characterised (Inwongwan et al., 2019). To complete the GEM of *E. gracilis*, identifying the subcellular locations and functions of all metabolic pathways would greatly flavour the improvement of the network.

As the limited availability of the data is one of the main challenges, all reported data is collated and present in this section (Table 1). As mentioned, the genome of *E. gracilis* has not been completely analysed; thus, reconstructing the GEM of *E. gracilis* is merely possible. A metabolic network model of *E. gracilis* was generated based on high quality transcriptomic data (O’Neill et al., 2015b; Yoshida et al., 2016) and is used in the same sense as GEM. The model was able to predict the metabolic fluxes of the central metabolic pathways during the heterotrophic metabolism of various carbon substrates, but the model has not been further developed or validated to predict the metabolic phenotypes in any other growth conditions (Inwongwan, 2021). However, this transcriptomic-based metabolic network model of *E. gracilis* shows a possibility to reconstruct a functioning metabolic network model without the complete genome data.

**Table 1:** Published transcriptome, organelle genome and proteome data of *E. gracilis*.

Analysis	Growth condition	Number of component	Reference
Transcriptome	High nutrient: EG + JM media with 15 gL^−1^ glucose, 30°C, 200 rpm, in the dark Low nutrient: CaCl_2_ (0.1 g L^−1^), NaOAc·(H_2_O)_3_ (1 g L^−1^) and JM medium with 15 gL^−1^ agar, 21°C, ambient light	32,128 unique protein-encoding genes	O’Neill et al. (2015b)
	Koren-Hutner medium, 26°C, 120 rpm, 100 μmol photons m^−2^ s^−1^, stationary phase cells were incubated in anaerobic condition for 5 min.	26,479 unique protein-encoding genes	Yoshida et al. (2016)
	Dark grown: Hutner medium, ambient temperature, in the dark Light grown: Hutner medium, ambient temperature, illumination from a 60-W tungsten filament bulb at 20 cm from the culture vessel	36,526 unique protein-encoding genes	Ebenezer et al. (2019)
	Liquid mineral medium tris-minimum-phosphate with vitamin mixture supplemented, pH 7.0, 25°C acetate (60 mM) added, in the dark acetate (60 mM) added, low PPFD (50 μE m^−2^ s^−1^) acetate (60 mM) added, medium PPFD (200 μE m^−2^ s^−1^) No acetate added, low PPFD (50 μE m^−2^ s^−1^)	49,922 unique protein-encoding genes	Cordoba et al. (2021)
	EG medium, pH 3.5, 21°C, 96 μmol photons m^−2^ s^−1^ for a photoperiod of 16 h light/8 h dark, control and 5 μmol L^−1^ Hg(NO_3_)_2_ treated	439,129 assembled genes	Mangal et al. (2022)
Chloroplast genome	Standard cultivation procedures, unspecified	55 annotated genes	Hallick et al. (1993)
Mitochondrial genome	Hutner medium, 27°C, constant shaking, permanent light conditions 10 μm/m^−2^ s^−1^	7 annotated genes	Dobáková et al. (2015)
Proteome	Dark grown: Hutner medium, ambient temperature, in the dark Light grown: Hutner medium, ambient temperature, illumination from a 60-W tungsten filament bulb at 20 cm from the culture vessel	8,661 proteins	Ebenezer et al. (2019)
	GNY medium, 23°C, 150 rpm, white light (2000 lx) for a photoperiod of 12 h light/12 h dark, heavy metals treated: mercury (as HgCl_2_), lead (as Pb(NO_3_)_2_) and cadmium (as CdCl_2_).	5,325 proteins	Khatiwada et al. (2020)
	Wild-type and Oflaxocin bleached strains, EM medium with 1% ethanol, 25°C, 50 μmol photons m^−2^ s^−1^	1,572 proteins	Chen et al. (2022)
Plastid proteome	Unspecified	1,345 proteins	Novák Vanclová et al. (2020)
Mitochondrial proteome	Non-phtosyntheisc mutant (strain SM-ZK), Koren–Hutner (KH) medium, 26°C, 120 rpm, under continuous light conditions of 50 μmol photons m^−2^ s^−1^	714 proteins	Tamaki et al. (2020)
	Hutner medium, 27°C, constant shaking, permanent light conditions 10 μm/m^−2^ s^−1^	2,704 proteins	Hammond et al. (2020)

The metabolic model reconstruction is an iterative process that should be continuously adjusted with the newly available data to improve the accuracy and completeness of the model. In the light of high throughput analysis of multiomics data, the extensive availability of transcriptome and proteome data could increase the reliability of the GEM of *E. gracilis*. Several approaches were developed to integrate the omics data with GEM. For example, based on seeking steady states of regulatory network, FlexFlux combine the analysis of regulatory networks with flux balance analysis (FBA) of GEM (Marmiesse et al., 2015), and Metabolic and Expression models (ME-models) includes metabolic and transcriptomic expression with the analysis of GEM (Lerman et al., 2012). Besides, the available transcriptome and proteome data of *E. gracilis* could be used to improve the draft reconstruction of the GEM. The transcriptomes of *E. gracilis* from various conditions provide potential metabolic capacity that could be used to draft a comprehensive GEM. The proteomes of *E. gracilis* provide further insights of the metabolic operation specific to the conditions of interest. These data could help refine the GEM to overcome the cryptic post-transcriptional regulation process. In addition, to constraint or validate the accuracy of the model, numerous extensive biochemical and physiological data of *E. gracilis* are required, such as biomass composition, growth characteristics, non-growth associated maintenance, carbon conversion efficiency, overall metabolic rate, metabolome and mitochondrial physiology. Some of these data are seldomly studied and reported, thus, the currently available data potentially contributing to the reconstruction of a complete GEM of *E. gracilis* might still be far from sufficient and will need to be further analysed.

## 4. Potential applications of the *Euglena* model in metabolic engineering

*Euglena*, as mentioned, is used to synthesise a number of high-value compounds (Gissibl et al., 2019). Though several bioproducts are commercially available, strain improvement to allow cost-competitive production is still important. Low production yield is one of the challenges that slows down the commercialisation of these natural products and this could be because the metabolic flux or the flow of metabolites to the desirable final products is low (Liu et al., 2017). Metabolic engineering is, therefore, of interest, as this approach could increase the flux towards desirable products in a stepwise manner by manipulating the expression of bottleneck genes. In order to do so, information regarding *Euglena* metabolic networks and bottleneck reactions towards the target products is essential to ensure successful engineering – which could be implemented by knocking out, overexpressing or heterologously expressing of particular genes. As the development of genetic engineering in *Euglena* has just been kicked off, not a lot of works have been published, and only a couple of reviews summarising the reported engineering tools are available (Harada et al., 2020; Khatiwada et al., 2020; Chen et al., 2022). The delayed development could be due to the lack of knowledge on their molecular characteristics, including their complete nuclear genome sequences. Moreover, they have distinct characteristics such as their chloroplasts that are surrounded with three enveloping membranes, which makes it challenging for DNA transformation. Cellular characteristics important for engineering are often addressed along with the development of compatible engineering tools. Antibiotic resistance is one of the important properties addressed when developing transformation techniques to identify suitable selectable markers (Khatiwada et al., 2019). A chloroplast transformation technique was developed in 2001 using biolistic transformation with confirmed transgene transcription (Doetsch et al., 2001). Later on, the focus was shifted to the nucleus, electroporation was developed and demonstrated to be a potential technique to transform fluorescent markers into the nucleus of *E. gracilis* (Ohmachi et al., 2016). Recently, a nuclear transformation technique with the help of *Agrobacterium* was also demonstrated to be successful in *E. gracilis* (Khatiwada et al., 2019; Becker et al., 2021). RNA interference (RNAi), a technique to suppress gene expression, was also investigated in *Euglena*. As a metabolic engineering strategy, a few reports have used RNAi to silence genes encoding enzymes in competitive pathways for natural product production in *Euglena* (Nakazawa et al., 2015; Kato et al., 2017; Kimura and Ishikawa, 2018). Even though the development of genetic engineering tools in *Euglena* has been relatively slow compared to other model organisms, the number of published works has gradually increased, including the first report of a groundbreaking tool, CRISPR, in 2019 (Nomura et al., 2020). This indicates increased attention towards *Euglena* as a potential host for genetic engineering and bioproduction, as the most recent development of CRISPR system in *Euglena* was to create a non-motile mutant to facilitate the harvesting process (Ishikawa et al., 2022).

As the number of genetic engineering toolkits for *Euglena* has been increasing over time, this ensures the feasibility to metabolically engineer them as a cell factory for attractive chemicals and with the help of metabolic network models, metabolic bottlenecks can be identified. To the best of our knowledge, model-assisted metabolic engineering in *Euglena* has yet to be reported. However, examples have been successfully demonstrated in other hosts, including *Escherichia coli* and *Saccharomyces cerevisiae*. To provide some examples, in 2018, a metabolic model for hydrocarbon production in *E. coli* was reconstructed, and flux balance analysis (FBA), was used to identify metabolic engineering strategies to increase the production of long-chain alkanes and alcohols (Fatma et al., 2018). Recently, a report also utilised FBA to force the flux towards *n*-butane in *E. coli*, which was found to vastly increase the production by 168 folds (Liu et al., 2022). Similarly, in the case of *S. cerevisiae*, FBA was used to identify the target to fine-tune central carbon metabolism to increase the levels of acetyl-CoA and malonyl-CoA (Ferreira et al., 2019). To provide more relatable examples, GEM has also been constructed in photosynthetic organisms. Several metabolic models of cyanobacteria (i.e., *Synechocystis* sp. PCC 6803, *Synechococcus* sp. PCC 7002 and *Arthrospira platensis*) have been reconstructed and summarised in a previous review (Santos-Merino et al., 2019). Several works have reported on the use model-assisted metabolic engineering to improve the production of bioproducts from cyanobacteria including limonene (Wang et al., 2016), 1,3-propanediol (Hirokawa et al., 2017), ethanol (Yoshikawa et al., 2017) and *n*-butanol (Anfelt et al., 2015). Compared to prokaryotic cells, the field of metabolic modeling in eukaryotic photosynthetic organisms has progressed slowly due to the complexity of their massive genome size and cellular compartmentation (Kruger and Ratcliffe, 2015). However, models of Arabidopsis and tomato, two model organisms, have been successfully constructed (Poolman et al., 2009; Yuan et al., 2016) and proven useful for predicting the metabolic phenotype of the organisms. In the case of eukaryotic microalgae, metabolic models for several microalgae, including *Chlorella* and *Chlamydomonas*, have been reconstructed (Chang et al., 2011; Kliphuis et al., 2012; Zuñiga et al., 2016; Tibocha-Bonilla et al., 2018). These models have mostly been used in order to understand the native metabolism of the microalgae. A recent study has reported the use of the metabolic model of *Chlorella vulgaris* to predict cultivating conditions for growth optimisation. Interestingly, the predicted conditions also led to increased production of fatty acid methyl ester (FAME) and lutein (Li et al., 2019), demonstrating the potential of using metabolic models to enhance bioproduction in microalgae. Redirection of flux from β-1,3-glucan biosynthetic pathway to other pathways could be an approach that would allow increased production of wide range chemicals in *Euglena*. To be specific, according to the flux maps of heterotrophic metabolism from ^13^C metabolic flux analysis, *Euglena* tends to direct glucose intake (37–41%) towards β-1,3-glucan storage (Inwongwan, 2021). To optimise the production of lipid production with this information, for example, down regulation of paramylon synthetase, an enzyme responsible for β-1,3-glucan synthesis from UDP-glucose, could be the potential strategy. Altogether, from a metabolic engineering point of view, it could be concluded that metabolic network models are valuable for rational design engineering.

## Conclusion

This mini-review has summarised the current state of metabolic network reconstruction in *E. gracilis* and the challenges that obstruct the progression of the model. Generating a definitive metabolic model of *E. gracilis* could significantly contribute to the application of this organism as a cell factory for production of valuable compounds. The model can be used to study the metabolism of *E. gracilis* in various conditions and to predict targets for metabolic engineering. Its potential has been demonstrated in other model organisms, yet model-assisted engineering has never been reported in *Euglena*. This could be because, apart from the unavailability completed model of *Euglena*, the delayed development of genetic engineering toolkits though rapid development has been observed over recent years. Moreover, in this work, we implicate potential applications of metabolic network reconstruction of *Euglena* through metabolic engineering. Overall, we anticipate that the use of model-assisted metabolic engineering in *Euglena* will increase in the near future.

## Author contributions

SI and PS were responsible for the main writing of this manuscript. All authors contributed to the article and approved the submitted version.

## Funding

This research work was partially supported by Chiang Mai University.

## Conflict of interest

The authors declare that the research was conducted in the absence of any commercial or financial relationships that could be construed as a potential conflict of interest.

## Publisher’s note

All claims expressed in this article are solely those of the authors and do not necessarily represent those of their affiliated organizations, or those of the publisher, the editors and the reviewers. Any product that may be evaluated in this article, or claim that may be made by its manufacturer, is not guaranteed or endorsed by the publisher.
